# Death of Dr. S. W. Butler

**Published:** 1874-01

**Authors:** 


*Death of Dr. S. W. Butler.*

It is with profound regret we learn, through the *Medical and Surgical Reporter*, of the death of one of its editors, Dr. S. W. Butler. Another generous, noble-hearted brother has gone, leaving the profession, he loved so well, the poorer by his untimely departure. Peace to his ashes!

